# Sequence analysis of *Drd2*, *Drd4*, and *Dat1 *in SHR and WKY rat strains

**DOI:** 10.1186/1744-9081-1-24

**Published:** 2005-12-15

**Authors:** Jonathan Mill, Terje Sagvolden, Philip Asherson

**Affiliations:** 1 MRC SGDP Centre, Institute of Psychiatry, London, UK; 2 Cell and Molecular Biology Division, Toronto Western Research Institute, University Health Network, Toronto, ON, Canada; 3 University of Oslo, Oslo, Norway

## Abstract

**Background:**

The Spontaneously Hypertensive Rat (SHR) shows a number of behaviours that closely parallel those seen in children with attention-deficit hyperactivity disorder. These include motor hyperactivity, excessive responses under a fixed-interval/extinction schedule, difficulty in acquiring operant tasks and increased sensitivity to immediate behavioural reinforcement. As in children with ADHD, the behavioural and cognitive deficits in the SHR are responsive to stimulants, including d-amphetamine and d,l-methylphenidate. The non-hyperactive Wistar Kyoto (WKY) rat strain is often used as a control in behavioural studies of the SHR, and WKY itself has been suggested to be a useful animal model of depression. Numerous studies have shown that dopaminergic neurotransmission is altered between the two strains. Human genetic studies have found associations between several dopaminergic genes and both ADHD and depression.

**Methods:**

We sequenced three candidate dopaminergic genes (*Drd2*, *Drd4*, and *Dat1*) in the SHR and WKY to identify between-strain sequence differences.

**Results:**

No between-strain sequence differences were found in either *Drd2 *or *Drd4*, but several variations were found in the *Dat1 *gene that encodes the dopamine transporter.

**Conclusion:**

It is plausible that DNA sequence changes in the *Dat1 *gene account for some of the behavioural differences observed between the SHR and WKY strains. Future work will focus on elucidating the functional effects of the observed polymorphisms.

## Background

Attention-deficit hyperactivity disorder (ADHD) is a common neurobehavioural disorder defined by symptoms of developmentally inappropriate inattention, impulsivity and hyperactivity. It is estimated that between 3–6% of school age children are diagnosed with ADHD, making it the most prevalent disorder of childhood. While the precise aetiology of ADHD is yet to be ascertained, it is clear from numerous family, twin and adoption studies that genetic factors play in a key role in susceptibility to the disorder. Polymorphisms in several genes have been associated with ADHD, with a particular focus on genes implicated in monoamine neurotransmission [1].

A number of animal models have been proposed for ADHD and these have helped inform research into the biological basis of the clinical disorder. The spontaneously hypertensive rat (SHR) is one of the most widely validated animal models of ADHD [2,3]. The SHR shows a number of behaviours that closely parallel those seen in children with ADHD including motor hyperactivity, increased impulsiveness and deficient sustained attention [3,4]. Furthermore, like children with ADHD, the SHR is more sensitive to immediate behavioural reinforcement and less sensitive to delayed reinforcement than non-hypertensive WKY control rats [3,4]. The behavioural and cognitive deficits in the SHR are responsive to stimulants, including d-amphetamine and d,l-methylphenidate [5]. Finally, several studies have shown that dopaminergic and noradrenergic neurotransmission is altered in the SHR compared to the WKY, strongly implicating these systems in the aetiology of ADHD [2,6,7].

The WKY strain, from which the SHR was initially derived by selective outbreeding [8], is itself proposed to be a model of another psychiatric condition – depression. As for ADHD, the aetiology of depression has been shown to be strongly influenced by genetic factors [9] and dysregulation of the dopaminergic system has been strongly implicated [10]. WKY rats have been shown to exhibit exaggerated neuroendocrine and behavioral responses to stress that exceed normal controls and are especially prone to develop stress-induced depressive disorder [11,12]. A recent study by Will et al found that selectively bred WKY rats were a particularly good animal model of depression and hyper-responsiveness to anti-depressants [13]. Interestingly, the dopamine neurotransmitter pathway has been strongly implicated in the depression-like behaviours exhibited by WKY rats. Jiao et al observed differences in the density and distribution of dopamine transporter sites in WKY rats that may lead to altered modulation of synaptic dopamine levels in the cell body and mesolimbic regions [14].

In this study we have sequenced three dopaminergic candidate genes (*Drd2, Drd4*, and *Dat1*) in the SHR and WKY rat strains to identify potential genetic variants that may explain some of the behavioural differences observed between the two strains.

## Methods

Blood was obtained from animals housed at the University of Oslo and DNA was extracted using a standard protocol [15]. Bioinformatic analyses were performed to identify regions in the rat genome containing homologues of human ADHD candidate genes using sequence data deposited in the Rat Genome Database . Where no annotated rat genome sequence was available, BLAST searches were performed on raw sequence data to identify the relevant regions . Primers were designed to span the promoter and exonic regions of three candidate genes (*Drd2*, *Drd4*, and *Dat1*) using Primer Express software (Applied Biosystems, Foster City, CA, USA). To conserve space oligo sequences are not given in this manuscript, but are available from the authors on request. The chromosomal location, amino-acid length and human-rat homology for the three genes sequenced in this study can be seen in Table 1.

The exonic regions were amplified on an MJ PTC-225 thermal cycler (MJ Research) with an initial 9-min denaturing step at 95°C, followed by 35 cycles of 93°C for 1 min, 55°C for 1 min and 72°C for 1 min, and a final extension phase of 72°C for 10 min. Reactions were performed in 22 ul volumes and included 50 ng of genomic DNA, 1.5 mM MgCl_2_, 0.2 mM dNTP's, 10 mM GeneAmp 10× PCR Gold Buffer (PE Applied Biosystems, Foster City, US) and 1 unit of AmpliTaq Gold (PE Applied Biosystems, Foster City, US). PCR products were run out on a 2% agarose gel stained with ethidium bromide and analysed under UV light. PCR products were purified using Qiagen Gel Extraction Columns (Qiagen, Crawley, UK). Following purification, forward and reverse dye terminator sequencing was carried out using ABI BigDye V3.0 and samples run on either an ABI 377 or 3100 machine (Applied Biosystems, Foster City, CA, USA). Sequencing traces were analysed using Sequencher software (GeneCodes Corporation, Ann Arbor, MI, USA), and multiple sequences aligned to aid mutation detection.

**Table 1:** The chromosomal location, amino-acid length and human-rat homology for the three genes sequenced in this study.

Gene	Human		Rat		Homology
ID	Location	AA	Location	AA	(%)
DRD2	11q23.2	443	8q23	444	95%
DRD4	11q15.5	387	1q41	385	75%
DAT1	5p15.33	620	1p11	619	94%

## Results

### Drd2

The rat *Drd2 *gene is located on chromosome 8q23 and is 95% homologous with the human *DRD2 *gene. The putative promoter and exonic regions of Drd2 were sequenced. No differences were observed between the SHR and WKY strains, and both sequences were identical to those in the Rat Genome Database .

### Drd4

The rat *Drd4 *gene is located on chromosome 1q41 and shares 75% homology with the human *DRD4 *gene. The putative promoter, exons, and introns of *Drd4 *were sequenced. No differences were found between the SHR and WKY strains, although both were found to contain non-coding sequence differences from the Brown Norway Rat sequence available in the Rat Genome Database (see Table 2). These differences included two single nucleotide polymorphisms (SNPs), one located in the putative 5' promoter region of the gene and the other in intron 2, along with length variation at a CA microsatellite repeat in intron 2. In addition, neither the WKY or SHR strains were found to have the short first intron (GGCGCG) present between exon 1 and 2 in the sequence available in the Rat Genome Database.

**Table 2:** Variants noted in the *Drd4 *gene in both WKY and SHR strains compared to the sequence deposited in the Rat Genome Database .

**Location**	**Variation**
Promoter region	GATGAA[G/T]AGTGAG*
Intron 1	GGCGCG (not present in WKY/SHR)
Intron 2	CACA (2 extra CA motifs in SHR/WKY)
Intron 2	GAATGG[A/G]GACATA*

*WKY/SHR allele given second.

### Dat1

**Figure 1:**
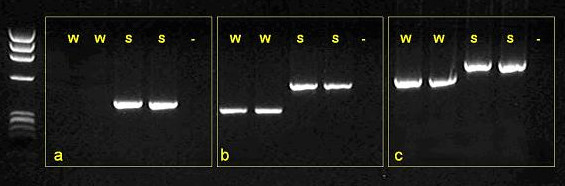
**PCR amplification of *Dat1 *exon 3 using three alternative primer pairs on SHR (S) and WKY (W) DNA**. On WKY DNA the reaction did not work using primer set (a) and resulted in a smaller PCR product than seen using SHR DNA with sets (b) and (c).

**Figure 2:**
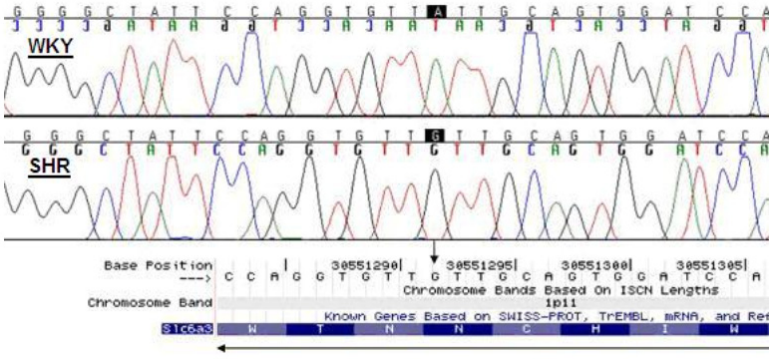
**Single base polymorphism in exon 3 of *Dat1*: WKY (T) → SHR (C)**. The polymorphism is silent, and in both strains results in anasparagine amino-acid.

The rat *Dat1 *gene is located on chromosome 1p11 and is 94% homologous with the human *DAT1 *gene. The putative promoter and exonic regions of *Dat1 *were sequenced. The exon 3 amplicon could not initially be amplified by PCR in the WKY strain (see Figure 1a). Subsequent PCR amplification using more distal primers suggested the presence of extra sequence in the SHR compared to the WKY (see Figures 1b and 1c). Sequencing of this region highlighted a synonymous single base change (T→C) within the coding sequence of exon 3 (see Figure 2), and a 160 bp section of sequence immediately upstream of exon 3 present in SHR but not WKY. Bioinformatic analysis of the public rat genome database demonstrated that this 160 bp sequence is also present ~1000 bp upstream (in intron 4 of *Dat1*).

## Discussion

In this study we sequenced three dopaminergic genes to examine differences between SHR and WKY rat strains. No between-strain sequence differences were found in genes encoding either the dopamine D2 receptor (*Drd2*) or the dopamine D4 receptor (*Drd4*), although for *Drd4 *both the WKY and SHR strains were found to differ from the sequence available in the Rat Genome Database . In contrast, several between-strain variations were found in the dopamine transporter gene (*Dat1*). Although none of the sequence changes results in a direct coding change to the DAT protein, it is plausible that they may alter expression-related processes such as transcription or splicing efficiency. Alternatively, it is possible that these changes are markers of other linked mutations carried on the same chromosome. These results are interesting given that the SHR and WKY strains are considered to be valid models of ADHD and depression respectively, and the postulated role of disrupted dopaminergic neurotransmission in both disorders.

It is pertinent that abnormalities in DAT expression and functioning have been noted in both rat strains. SHR strains have been shown to exhibit elevated DAT expression in mesocortical projections [16,17]. It appears that excess DAT expression in the SHR may not be directly genetic in origin, but is in fact a response to excess mesocortical dopamine during early development resulting from hypofunctioning DAT protein that *is *presumably genetic [2,16]. The WKY strain also appears to have an unusual DAT profile compared to non-depressive control strains. Jiao et al report lower DAT density in the nucleus accumbens, amygdala, ventral tegmental area, and the reticular part of the substantia nigra of these animals, but higher expression in the hippocampus and hypothalamus [14].

It is interesting that these findings are partially mirrored in studies on human psychiatric patients. Whilst individuals with ADHD have been shown to exhibit increased DAT density in the brain [18,19], depressive patients were found to have overall decreased levels of DAT [20]. Furthermore, genetic association studies suggests an association between a polymorphism in the human dopamine transporter gene (*DAT1*) and ADHD [21], although to date there is no evidence linking this polymorphism to the aetiology of depression.

## Conclusion

In this study we have sequenced three dopaminergic genes in two inbred rat strains considered to be good models of human psychiatric illness. No between strain differences were observed in either the *Drd2 *or *Drd4 *genes, suggesting that neither gene is likely to mediate the behavioural differences observed between the WKY and SHR strains, although a number of polymorphisms common to both strains were detected in *Drd4*. In contrast, WKY/SHR differences were observed in the 3^rd ^exon of *Dat1*. Whilst these mutations do not result in direct amino-acid changes to the DAT protein, it is possible that they mediate some other process that explains the differences in DAT expression and function observed between the two strains. Future work should focus on further characterizing the genetic differences between these two strains, and investigating the functional consequences of the observed polymorphisms and how they relate to the putative depressive and hyperactive behaviours observed in the two strains.

## Competing interests

The author(s) declare they have no competing interests.

## Authors' contributions

JM carried out the molecular genetic work and drafted the manuscript. TS provided the animal tissue used in this study and participated in the overall study design. PA supervised the project and helped draft the manuscript. All authors read and approved the final manuscript.
